# How to package the RNA of HIV-1

**DOI:** 10.7554/eLife.63585

**Published:** 2020-11-17

**Authors:** Alex Kleinpeter, Eric O Freed

**Affiliations:** HIV Dynamics and Replication Program, Center for Cancer Research, National Cancer InstituteFrederickUnited States

**Keywords:** HIV-1, integrase, maturation, integrase-RNA interactions, viral maturation, Human, Virus

## Abstract

Interactions between viral RNA and the integrase enzyme are required for HIV-1 particles to become infectious, a process that can be disrupted through multiple mechanisms.

**Related research article** Elliott JL, Eschbach JE, Koneru PC, Li W, Puray-Chavez M, Townsend D, Lawson DQ, Engelman AN, Kvaratskhelia M, Kutluay SB. 2020. Integrase-RNA interactions underscore the critical role of integrase in HIV-1 virion morphogenesis. *eLife* **9**:e54311. doi: 10.7554/eLife.54311

Virus particles rely on host cells to replicate and infect other cells. Key steps in this process include entry into the host cell, gene expression, and the production of new viral particles. For some viruses, including HIV-1, this last step starts with the assembly of immature, non-infectious virus particles; the complex process by which these particles mature is not fully understood.

Successful HIV-1 maturation culminates in the assembly of a conical-shaped core structure called the capsid, which encloses the viral RNA (vRNA) as well as two viral enzymes: reverse transcriptase, which produces DNA from vRNA, and integrase, which is best known for catalyzing the integration of vDNA into the genome of the host. It was shown many years ago that some deletions in the gene that codes for integrase result in a curious, eccentric core phenotype: empty capsids are formed and an electron-dense material, which is presumed to contain the vRNA, is present outside of the capsid (Engelman et al., 1995; Figure 1). This phenotype is associated with defects in multiple steps of the virus replication cycle, including reverse transcription.

**Figure 1.**
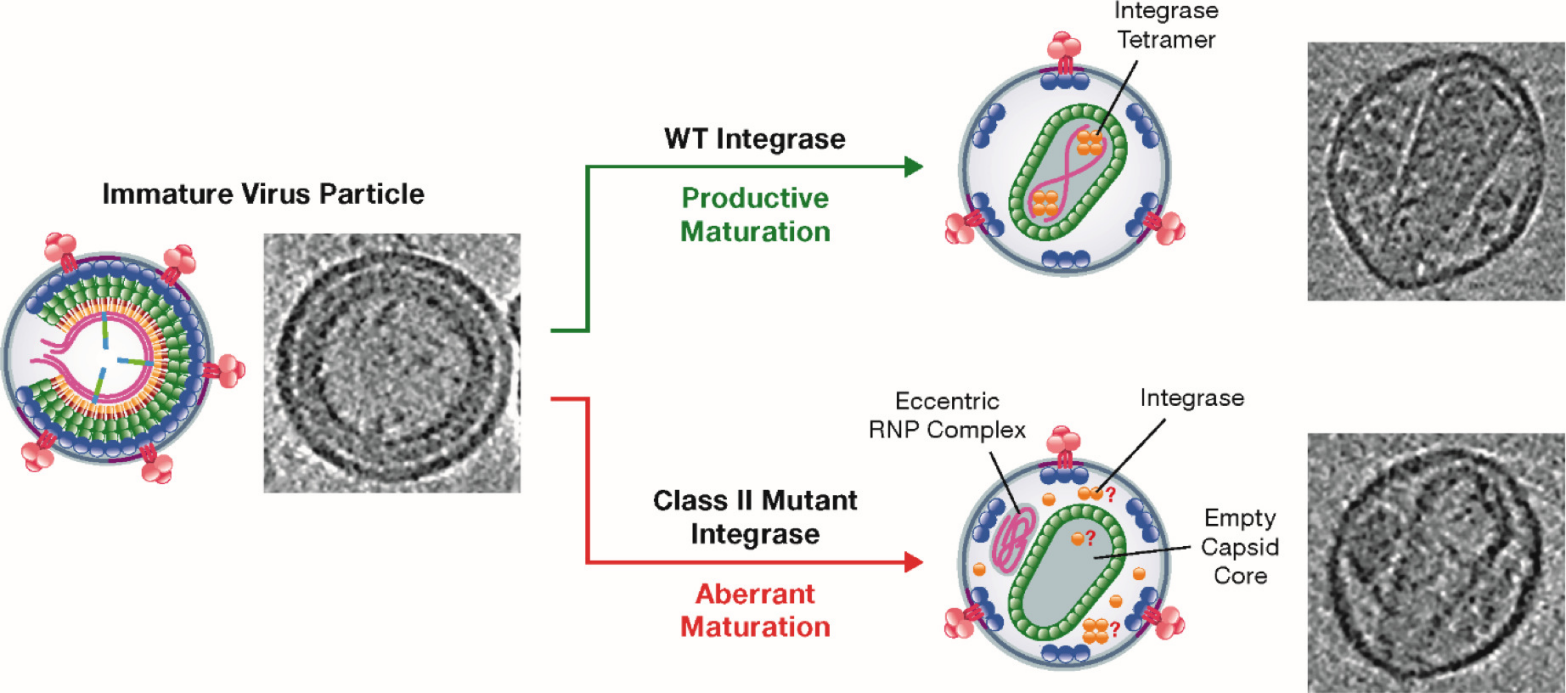
Class II mutant viral integrases and eccentric core formation. HIV-1 initially assembles into an immature virus particle (left; schematic diagram and electron micrograph), which subsequently undergoes a series of changes that result in the assembly of a mature capsid core (top right). Wild-type integrase (yellow circles) plays a key role during maturation by forming tetramers that interact with the viral RNA (pink strands) and ensure that it is packaged inside the capsid. Class II mutant integrases cause aberrant maturation (bottom right) due to direct or indirect loss of the interaction between the enzyme and the viral RNA: this results in the production of an eccentric complex containing the viral RNA outside the capsid. In these eccentric particles, the location of the integrases and whether they form tetramers is not known.

Image credit: Figure modified from Kleinpeter and Freed, 2020 (CC BY 4.0).

Following this discovery, numerous integrase mutants – known as class II mutants – were shown to display a similar wide-ranging phenotype (Jurado et al., 2013; Quillent et al., 1996). Multiple studies have suggested that these class II mutants have defects in reverse transcription, possibly because they have lost their vRNA, or because reverse transcriptase becomes physically separated from the genetic information upon infection of a target cell (Koneru et al., 2019; Madison et al., 2017). Now, in eLife, Sebla Kutluay of Washington University in St. Louis and colleagues – including Jennifer Elliot as first author – report how class II integrase substitutions impair the maturation of HIV-1 particles (Elliott et al., 2020).

First, Elliott et al. confirmed a previous observation: that the binding of integrase to vRNA may underpin its role during HIV-1 maturation (Kessl et al., 2016). The team examined replication defects induced by a panel of more than 25 class II integrase substitutions and, as expected, particle infectivity and reverse transcription products dropped in all mutants. Moreover, the class II substitutions disrupted the interactions between integrase and vRNA in three distinct ways.

For one subset of substitutions, the levels of integrase in both cells and virus particles were significantly decreased, suggesting that these mutations prevent the expression of integrase or its packaging inside new virus particles. The other two subsets involved the disruption of integrase binding to vRNA, rather than the packaging of the enzyme into the particles. Interestingly, many substitutions involve integrase residues outside the region previously implicated in RNA binding (Kessl et al., 2016). The second subset of mutations resulted in the interaction between the enzyme and vRNA being directly blocked, while the third subset led to interactions between integrase enzymes being impaired. In particular, fewer enzymes were able to form tetramers, the structures containing four copies of integrase that normally bind to vRNA.

Finally, Elliott et al. confirmed that the eccentric core phenotype was present in each of the mutants, and they observed lower amounts of vRNA in target cells just after infection. This suggests that without the protection of the capsid shell, exposure to the intracellular environment decreases the stability of the vRNA.

The recent discovery of a new class of HIV-1 inhibitors, known as ALLINIs (allosteric integrase inhibitors), is making these results particularly relevant (reviewed in Elliott and Kutluay, 2020; Engelman, 2019; Kleinpeter and Freed, 2020). These compounds induce aberrant integrase multimerization and therefore disrupt the binding of the enzyme to vRNA, resulting in particles with eccentric cores reminiscent of those found in class II mutants. Further characterization of these mutants, and of the role of integrase during HIV-1 maturation, may help in the development of ALLINIs as potential HIV-1 therapeutics.

This work also raises several questions. First, it is still unclear whether the integrase only helps the vRNA to be packaged into the capsid, or if it also participates in the construction of the capsid itself. While Elliott et al. show that capsids assembled in particles with mutant integrase are as stable as those assembled with the normal version of the enzyme, it was previously reported that ALLINIs and a class II integrase mutation disrupt the formation of the capsid (Fontana et al., 2015). Second, what happens to integrase when ‘empty’ capsids are assembled? The experiments by Elliott et al. reveal that mutant integrase enzymes, while physically associated with the capsid, are rapidly degraded after infection – potentially because they are located, unprotected, on the outside of the capsid. This suggests that in the absence of an interaction between integrase and vRNA, neither the enzyme nor the genetic material is packaged into the capsid, raising further questions about the molecular mechanisms driving packaging. Next, the fact that ALLINIs act by inducing aberrant integrase multimerization suggests that other classes of small molecules could be developed, which interfere with the packaging of vRNA into the capsid through different mechanisms. Finally, it remains unclear whether integrase enzymes in viruses related to HIV-1 also promote vRNA packaging into the capsid during maturation. Answering these questions will allow a greater understanding of how HIV-1 and related viruses mature, with implications for basic biology and new drug development.
